# The Outcomes of Superior Cavopulmonary Connection Operation: a Single Center Experience

**DOI:** 10.21470/1678-9741-2017-0025

**Published:** 2017

**Authors:** Alwaleed Al-Dairy, Maziar Gholampour Dehaki, Gholamreza Omrani, Ali Sadeghpour, Amir Hossein Jalali, Reza Sadat Afjehi, Mohammad Mahdavi, Mahmood Salesi

**Affiliations:** 1 Department of Cardiovascular Surgery, Division of Congenital Cardiac Surgery of Rajaie Cardiovascular Medical and Research Center, Iran University of Medical Sciences, Tehran, Iran.; 2 Department of Pediatric Cardiology, Rajaie Cardiovascular Medical and Research Center, Iran University of Medical Sciences, Tehran, Iran.; 3 Atherosclerosis Research Center, Baqiyatallah University of Medical Sciences, Tehran, Iran.

**Keywords:** Fontan Procedure, Heart Bypass, Right, Heart Ventricles/pathology, Heart Defects, Congenital/surgery

## Abstract

**Introduction:**

The superior cavopulmonary connection operation is one of the stages of the palliative surgical management for patients with functionally single ventricle. After surviving this stage, the patients are potential candidates for the final palliative procedure: the Fontan operation.

**Objectives:**

This study aimed to analyze the outcomes of superior cavopulmonary connection operations in our center and to identify factors affecting the survival and the progression to Fontan stage.

**Methods:**

The outcomes of 161 patients were retrospectively analyzed after undergoing superior cavopulmonary connection operation in our center between 2005 and 2015.

**Results:**

The early mortality rate was 2.5%. Five (3.1%) patients underwent takedown of the superior cavopulmonary connection. The rate of exclusion from the Fontan stage was 8.3%. Statistical analysis revealed that elevated mean pulmonary artery pressure preoperatively and the prior palliation with pulmonary artery banding were risk factors for both early mortality and takedown; however, the age, the morphology of the single ventricle and the type of operation were not considered risk factors.

**Conclusion:**

The superior cavopulmonary connection operation can be performed with low rate mortality and morbidity; however, the elevated mean pulmonary artery pressure preoperatively and the prior pulmonary artery banding are associated with poor outcomes.

**Table:** 

Abbreviations, acronyms & symbols	
CPB	= Cardiopulmonary bypass
CTA	= Computed tomographic angiography
LV	= Left ventricle
mPAP	= Mean pulmonary artery pressure
PAB	= Pulmonary artery banding
PAP	= Pulmonary artery pressure
RV	= Right ventricle
SCPC	= Superior cavopulmonary connection
TAPVC	= Total anomalous pulmonary venous connection
TCPC	= Total cavopulmonary connection
TTE	= Transthoracic echocardiography

## INTRODUCTION

The superior cavopulmonary connection (SCPC) operation represents one of the stages for the surgical palliation in patients with functionally univentricular hearts. This operation may or may not be preceded by a first stage palliation; however, it is well known that this operation results in more efficient oxygenation than the systemic pulmonary shunt with the advantage of avoiding the volume or pressure overload of the single ventricle^[1]^. There are two basic surgical techniques for creating a cavopulmonary connection, the bidirectional superior cavopulmonary anastomosis (bidirectional Glenn operation) and the Hemi-Fontan operation. In those patients who have an interruption of the inferior vena cava with azygous or hemiazygous continuation, a bilateral superior cavopulmonary connection operation is performed with all the systemic venous return is directed to the pulmonary circulation except for the portal venous return, this operation is called "Kawashima operation"^[2]^. However, the development of pulmonary arteriovenous malformations and pulmonary arteriovenous fistulae remains a potential complication following Kawashima operation^[2-6]^. The patients who survive the SCPC operation are potential candidates for the final palliative procedure: the Fontan operation^[7]^.

This study aimed to analyze the outcomes of SCPC operations in our center and to identify factors affecting the survival and the progression to Fontan stage.

## METHODS

### Study Protocol and Population

Between 2005 and 2015, 161 patients with single ventricle physiology due to variable congenital heart defects underwent SCPC in our center, Rajaie Cardiovascular Medical and Research Center. In a retrospective study, the outcomes of these patients concerning the clinical conditions, the survival rates, and the progression to the final palliative stage were analyzed (Fontan stage). Baseline demographics, preoperative, and intraoperative data were collected from their charts. This study protocol was approved by the local ethics committee in our institution.

### Patients Follow-Up

The patients were regularly followed up in the outpatient clinic (1 week and 1 month after surgery, then every 3 months), with complete physical examination and transthoracic echocardiography (TTE). The follow-up data were obtained from chart review, with special attention to survival and the completeness of the final palliative stage.

### Diagnostic Evaluations

The main diagnostic device was the TTE for both preoperative diagnosis and postoperative follow-up. For further anatomical evaluation and especially for measuring the mean pulmonary artery pressure (mPAP), cardiac catheterization was performed preoperatively in 113 (70%) patients. For those patients who had not undergone cardiac catheterization, the PAP was measured intraoperatively. Additionally, computed tomographic angiography (CTA) was performed in 90 (55.9%) patients.

### Statistical Analysis

Continuous variable were presented as mean ± SD or median (interquartile range) as appropriate. Qualitative variables were presented as frequency and percentage. Mann Whitney U test was used to compare two groups' means and *P* value < 0.05 was considered statistically significant. All statistical analyses were performed using SPSS 20 for windows (IBM Inc., Somers, NY, USA).

## RESULTS

### Baseline Characteristics

Median age at SCPC operation was 5±4.9 years (range 9 months to 24.5 years), and 54% of the patients were male (87 patients). Mean mPAP preoperatively was 13±3.6 mmHg (range 7-27 mmHg). The most common congenital heart defect in our patients was tricuspid atresia (60 patients, 37.3%). The underlying congenital heart defects are summarized in Table 1.

**Table 1:** The underlying congenital heart defects.

Congenital heart defect	Values^a^
TA	60 (37.3%)
PS or PA with or without VSD	23 (14.3%)
TGA	22(13.7%)
cc-TGA	11 (6.8%)
DILV	10 (6.2%)
Mitral atresia	9 (5.6%)
DORV or DOLV with upstairs downstairs ventricles	8 (5%)
Heterotaxy syndrome	7 (4.3%)
Unbalanced CAVSD	6 (3.7%)
Large multiple VSDs	5 (3.1%)

a All values are presented as number (%).

CAVSD=complete atrioventricular septal defect; cc-TGA=congenitally corrected transposition of great arteries; DILV= double inlet left ventricle; DOLV=double outlet left ventricle; DORV=double outlet right ventricle; PA=pulmonary atresia; PS=pulmonary stenosis; TA=tricuspid atresia; TGA=transposition of great arteries; VSD=ventricular septal defect

### Intra- and Post-Operative Outcomes

**Fig. 1:**
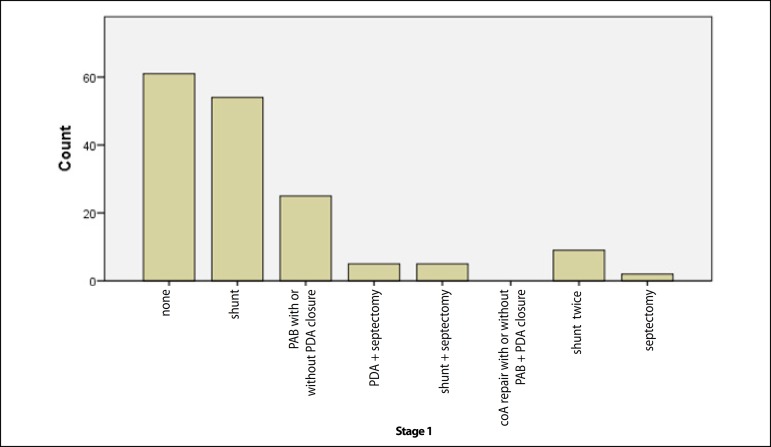
The palliative operations performed as first stage palliation. "Count" is expressed as absolute numbers. coA=Coarctation of the aorta; PAB=pulmonary artery banding; PDA=patent ductus arteriosus

Primary SCPC defined as SCPC operation without any previous palliative operations was performed in 61 (37.9%) patients, and secondary SCPC (with prior palliation) in the remainder. The prior palliative operations included systemic pulmonary shunt in 63 (39.1%) patients, pulmonary artery banding (PAB) in 25 (15.5%), PAB with atrial septectomy in 5 (3.1%), systemic pulmonary shunt with atrial septectomy in 5 (3.1%), and atrial septectomy in two (1.3%) (Figure 1).

The predominant ventricle was with left ventricle (LV) morphology in 118 (73.3%) patients, with right ventricle (RV) morphology in 41 (25.5%), and with intermediate morphology in two (1.2%).

**Fig. 2:**
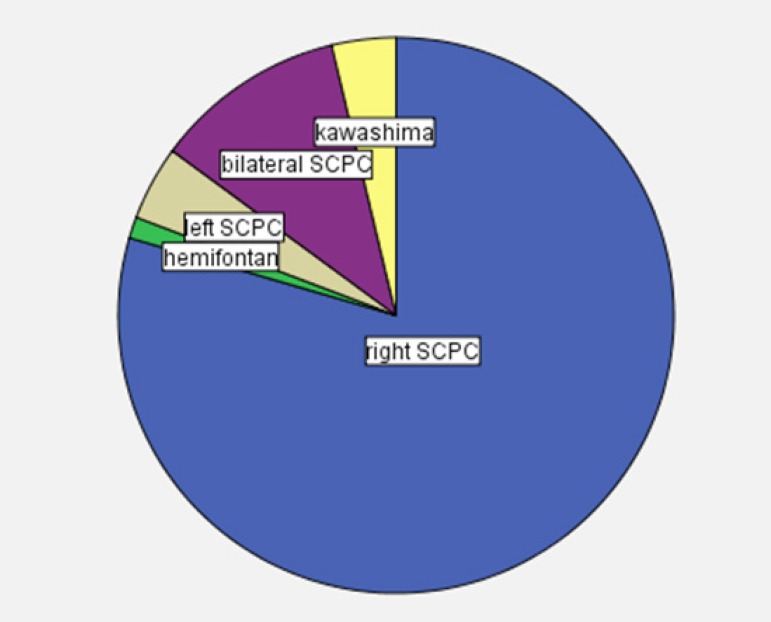
Type of superior cavopulmonary connection. SCPC=superior cavopulmonary connection. Right SCPC in 79.5% of the patients, left SCPC in 4.3%, bilateral SCPC in 11.2%, hemi-Fontan in 1.2%, and Kawashima operation in 3.8%.

The type of the SCPC operation was right SCPC in 128 (79.5%) patients, left SCPC in seven (4.3%), bilateral SCPC in 18 (11.2%), hemi-Fontan in two (1.2%), and Kawashima operation in six (3.8%) (Figure 2). The operation was carried out using cardiopulmonary bypass (CPB) except for 22 patients in whom right SCPC was performed without CPB (13.7% of the cohort). The azygous (or the hemiazygous) vein was ligated and divided in 96 (59.6%) patients. Previous systemic pulmonary shunt (if existed) was taken down in 75% of the cases, without any effect on the outcomes. Concomitant operations at the time of SCPC included: repair of pulmonary artery branches (n=8), atrioventricular valve repair (n=3), total anomalous pulmonary venous connection (TAPVC) repair (n=2), and atrial septectomy (n=2).

Four (2.5%) patients died in the hospital due to pulmonary infection (two patients), failure of the SCPC which was taken down (one patient), and low cardiac output syndrome with disseminated intra vascular coagulation (one patient). The characteristics of these patients are summarized in Table 2. Mean mPAP in this group of patients (in-hospital mortality) was 20±1.63 mmHg, which was significantly higher than that in the survived patients (12.85±3.44 mmHg), (*P*=0.001). Two patients underwent takedown of the SCPC on the same day of operation; one of them died in the hospital and the other was alive after a 2-year follow-up period. Twelve (7.5%) patients suffered from prolonged pleural effusion (> 14 days), with three of them having chylothorax.

**Table 2:** Characteristics of the in-hospital mortality patients.

Patient	Age^a^	Diagnosis	Prior palliation	Type of SCPC	mPAP^b^	Associated procedures
1	3	PS without VSD	Shunt	Right SCPC without pump	18	None
2	6	Unbalanced CAVSD	PAB	Right SCPC with pump	20	None
3	1.5	TA+PS	None	Right SCPC with pump	20	PA branch repair^c^
4	1	Heterotaxy syndrome	None	Right SCPC with pump	22	TAPVC repair

a age at operation in years,

b mean pulmonary artery pressure in mmHg preoperatively,

c pulmonary artery branch repair

CAVSD=complete atrioventricular septal defect; mPAP=mean pulmonary artery pressure; PA=pulmonary atresia; PAB=pulmonary artery banding; PS=pulmonary stenosis; SCPC=superior cavopulmonary connection; VSD=ventricular septal defect; TA=tricuspid atresia; TAPVC=total anomalous pulmonary venous connection

### Follow-Up

Median follow-up time after the SCPC operation was 3.1±1.9 years (range 6 months to 10 years). Two (1.27%) late deaths occurred during the follow-up period, both of them due to heart failure. The rate of freedom from mortality in the follow-up period was 96.27%. Thirty-seven (23.57%) patients underwent total cavopulmonary connection (TCPC), and 99 others (63%) are waiting for TCPC. Thirteen (8.3%) patients were not candidates for TCPC due to high PAP (7 patients of whom three patients underwent takedown of the SCPC), poor development of pulmonary arteries (three patients), ventricular dysfunction (two patients), and viral hepatitis (one patient). No patient (especially from those who underwent Kawashima operations) developed pulmonary arteriovenous fistulas during the period of this study.

### Takedown of the SCPC

Five patients underwent takedown of the SCPC (two on the same day of SCPC operation of whom one died, and three during follow-up). The common denominator among these patients was the prior palliation with PAB. Furthermore, their mean mPAP preoperatively (17.4±3.29 mmHg) was significantly elevated when compared with that of the other patients (12.87±3.5 mmHg), (*P*=0.01). All the patients who survived the takedown of the SCPC were excluded from the completeness of TCPC due to elevated mPAP. The characteristics of the patients who underwent takedown of the SCPC are summarized in Table 3.

**Table 3:** Characteristics of the takedown patients.

Patient	Age^a^	Diagnosis	Prior palliation	mPAP^b^	Time of takedown	Follow-up
1	1.25	Large multiple VSDs	PAB	12	The same operation day	2 years
2	6	Unbalanced CAVSD	PAB	20	The same operation day	Died in the hospital
3	3	DORV upstairs/downstairs ventricles	PAB	20	After 3.5 years	9 years
4	1	DORV upstairs/downstairs ventricles	PAB	17	After 3 years	9 years
5	2	DOLV upstairs/downstairs ventricles	PAB	18	After 3 years	7 years

a age at SCPC operation in years,

b mean pulmonary artery pressure in mmHg preoperatively.

CAVSD=complete atrioventricular septal defect; DORV=double outlet right ventricle; mPAP=mean pulmonary artery pressure; PA=pulmonary atresia; PAB=pulmonary artery banding; PS=pulmonary stenosis; SCPC=superior cavopulmonary connection; VSD=ventricular septal defect; TA=tricuspid atresia; TAPVC=total anomalous pulmonary venous connection

## DISCUSSION

The early mortality rate after SCPC operation in our study was 2.5%. Five (3.1%) patients underwent takedown of the SCPC of whom two at the same operation day and three later during the follow-up period. The rate of exclusion from the TCPC was 8.3%. Statistical analysis revealed that elevated mPAP preoperatively and the prior palliation with PAB were risk factors for both early mortality and takedown of the SCPC; however, the age, the morphology of the single ventricle, and the type of SCPC were not considered risk factors. The diagnosis of large multiple ventricular septal defects or the upstairs downstairs ventricles with double outlet right ventricle or double outlet left ventricle was associated with poor outcomes but due to the small number of patients a statistically significant correlation could not be found.

Preoperative mPAP has been reported as a risk factor for death after the Glenn procedure^[8]^, and mortality in those receiving pulmonary artery banding was high^[9]^, and these findings were compatible with ours. From our perspective it is essential to protect the pulmonary vascularity in patients with single ventricle and unrestricted pulmonary blood flow since that the PAP importantly affects the results of the surgical palliation in these patients.

There is no consensus regarding the ideal time for performing the SCPC in patients with single ventricle^[10]^ . Age did not seem to influence the outcomes; however, we recommend surgery as earlier as possible, although other logistic factors such as the availability of specialized centers and physicians may affect the trend to perform the SCPC earlier.

The elimination of an accessory pulmonary blood flow (prior systemic pulmonary shunt) at the time of SCPC operation did not affect the outcomes. On the other hand, some studies suggested that this may be advantageous on a long-term basis^[11]^.

Pulmonary arteriovenous malformations and pulmonary arteriovenous fistulas did not develop during the follow-up period in this study in patients who underwent Kawashima operation. In one study, this complication arose in 58% of the patients in a median follow-up period of 5 years after Kawashima operation^[6]^.

### Limitation

The retrospective nature of this study is one of its main limitations, and the short follow-up period in some patients was another considerable one.

## CONCLUSION

The SCPC operation is an essential stage for the surgical palliation in patients with univentricular heart and can be performed with a low rate of mortality and morbidity; however, the elevated mPAP and the prior palliation by PAB remain an important risk factors for poor outcomes.

**Table:** 

Authors' roles & responsibilities	
AAD	Substantial contributions to the conception or design of the work; final approval of the version to be published
MGD	Final approval of the version to be published
GO	Final approval of the version to be published
AS	Drafting the work or revising it critically for important intellectual content; final approval of the version to be published
AHJ	Drafting the work or revising it critically for important intellectual content; final approval of the version to be published
RSA	Drafting the work or revising it critically for important intellectual content; final approval of the version to be published
MM	Final approval of the version to be published
MS	Acquisition, analysis, or interpretation of data for the work; final approval of the version to be published
